# Infective Endocarditis Complicating Hypertrophic Obstructive Cardiomyopathy: Is Antibiotic Prophylaxis Really Unnecessary?

**DOI:** 10.2174/1573403X09666131202142312

**Published:** 2013-11

**Authors:** Ahmet Guler, Soe M. Aung, Beytullah Cakal, Can Y. Karabay, Yeliz Guler, Cevat Kirma

**Affiliations:** 1 Kosuyolu Heart Education and Research Hospital, Cardiology Clinic, Istanbul-TR; 2 Fatih University Faculty of Medicine, Department of Cardiology, Istanbul-TR

**Keywords:** Infective endocarditis, hypertrophic cardiomyopathy, prophylaxis.

## Abstract

Infective endocarditis is a relatively rare complication of hypertrophic cardiomyopathy. Infective endocarditis in hypertrophic cardiomyopathy is almost always seen in patients with outflow obstruction and is more common in those with both outflow obstruction and atrial dilatation. We present a case of culture negative mitral valve endocarditis in a previously asymptomatic woman with hypertrophic cardiomyopathy who died in the course of the disease.

## INTRODUCTION

Antibiotic prophylaxis is no longer recommended in patients with hypertrophic cardiomyopathy (HCM) in current guidelines unless otherwise indicated. We present a case of endocarditis in a patient with hypertrophic cardiomyopathy with significant outflow obstruction, and intend to discuss the use of antibiotic prophylaxis in such patients.

## CASE PRESENTATION

A 37-year-old woman was admitted due to a week-long history of malaise, myalgia, sweating and high fever. On admission, she had a body temperature of 39°C, a heart rate of 100 beats/ min and a blood pressure of 110/80 mmHg. The auscultation revealed a systolic murmur suggestive of mitral regurgitation. A 12-lead electrocardiogram showed a sinus tachycardia of 100 beats /min, signs of left ventricular hypertrophy and abnormal lateral repolarization. The chest X-ray showed a normal cardiothoracic index. Laboratory investigation revealed leukocytosis (13.700 /mm3) and raised inflammatory markers.

Transthoracic echocardiography (TTE) demonstrated minimally pericardial effusion, septal hypertrophy (wall thickness: 1.7cm) with systolic anterior motion of the mitral valve, left atrial enlargement (47 mm), mild to moderate mitral regurgitation and a suspected image of vegetation on the anterior mitral leaflet* (Fig. (**1**) and supplementary video 1).* A gradient of 130 mmHg was measured with continuous wave Doppler in the outflow tract*. *Transesophageal echocardiography showed vegetation on both atrial and ventricular aspects of the mitral anterior leaflet and mild to moderate mitral insufficiency *(Fig. (**2**) and supplementary video 2)*. On detailed examination of her medical history, it was learned that she had a tooth extraction 3 weeks ago and no antibiotic prophylaxis was given. The empirical antibiotherapy (aqueous crystalline penicillin G and gentamicin sulfate) was started after blood samples were drawn for cultures. On the second day of hospitalization, she was entubated as she developed severe dyspnea and hypotension. The bedside TTE revealed moderate to severe mitral regurgitation. The patient died of cardiac arrest subsequently before the planned surgery could be carried out. The patient’s blood cultures were later found to be negative. 

## DISCUSSION

Infective endocarditis (IE), although not very common, has been reported in HCM patients in the literature. The data relating the prevalence of IE are limited, and the most comprehensive study of prevalence and incidence was done by Spirito and colleagues [1]. They followed 810 patients, and the incidence of IE was reported to be 1.4 per 1000 person/year. The major risk factors were found to be outflow tract obstruction and left atrial dilatation. The underlying pathogenesis of IE in patients with HCM could be explained by the occurrence of endocardial damage secondary to the turbulent blood flow during ejection and the contact between the mitral anterior leaflet and the septum during systole [1-3]. Consequently, the vegetations were most commonly found on the ventricular aspect of anterior mitral leaflet.

Some significant changes have been made relating the antibiotic prophylaxis in recent guidelines [4, 5]. The reasons for changes could be summarized as follow; (1) bacteraemia could be caused by daily routine activities such as tooth brushing, flossing or chewing, (2) the lack of scientific evidence for the efficacy of IE prophylaxis, and (3) the possible emergence of resistant microorganisms and the risk of anaphylaxis. Unfortunately, decisions regarding antibiotic prophylaxis are being made without evidence from prospective, randomized, controlled trials. As for HCM patients, data are even more limited, and a definite decision regarding the prophylaxis could not be justified easily. Especially in patients with significant outflow tract obstruction, endothelial damage by turbulent flow and subsequent IE is likely, and further consideration should be made relating the prescription of antibiotic prophylaxis for prevention of this deadly complication. In conclusion, scientific evidence for antibiotic prophylaxis against IE is lacking for both HCM and other patient population, and more studies are needed.

## Figures and Tables

**Fig. (1):**
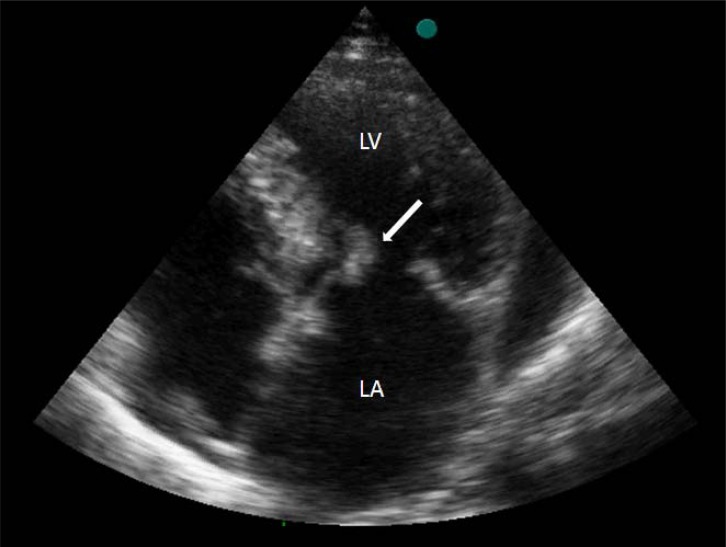
Transthoracic apical four-chamber view showing vegetation (arrow) on mitral anterior leaflet (LA: left atrium, LV: left ventricle).

**Fig. (2):**
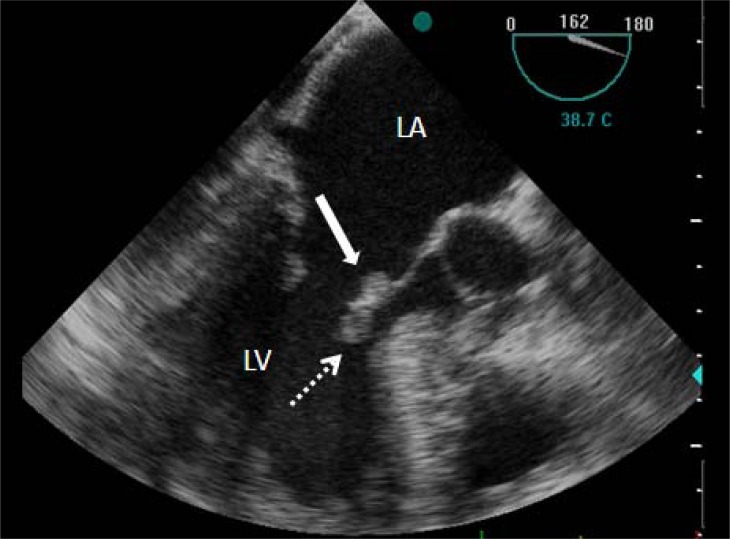
Transesophageal modified long-axis view showing vegetations on both atrial (arrow) and ventricular aspects (dotted arrow) of the mitral anterior leaflet (LA: left atrium, LV: left ventricle).
